# Relationships of orientation discrimination threshold and visual acuity with macular lesions in age-related macular degeneration

**DOI:** 10.1371/journal.pone.0185070

**Published:** 2017-09-18

**Authors:** Haojie Fu, Bin Zhang, Jianliang Tong, Harold Bedell, Hecheng Zhang, Yating Yang, Chaochao Nie, Yingdong Luo, Xiaoling Liu

**Affiliations:** 1 School of Ophthalmology and Optometry, Wenzhou Medical University, Wenzhou, Zhejiang, China; 2 College of Optometry, Nova Southeastern University, Davie, Florida, United States of America; 3 Brain Trauma Foundation, New York, New York, United States of America; 4 College of Optometry, University of Houston, Houston, Texas, United States of America; Save Sight Institute, AUSTRALIA

## Abstract

**Purpose:**

To measure visual acuity and metamorphopsia in patients with age-related macular degeneration (AMD) and to explore their relationship with macular lesions.

**Methods:**

In this cross-sectional study, a total of 32 normal subjects (32 eyes) and 35 AMD patients (35 eyes) were recruited. They were categorized into 4 groups: normal, dry AMD, non-active wet AMD, and active wet AMD. Best-corrected visual acuity (BCVA) was measured using the Early Treatment Diabetic Retinopathy Study protocol. Metamorphopsia was quantified with the orientation discrimination threshold (ODT). Macular lesions, including drusen, sub-retinal fluid (SRF), intra-retinal fluid (IRF), pigmented epithelium detachment (PED), and scarring, were identified with spectral-domain optical coherence tomography (SD-OCT). A linear regression model was established to identify the relationships between the functional and structural changes.

**Results:**

BCVA progressively worsened across the normal, dry AMD, non-active wet AMD, and active wet AMD groups (P < 0.001), and ODT increased across the groups (P < 0.001). The correlation between BCVA and ODT varied among the groups. The partial correlation between BCVA and ODT was −0.61 (P < 0.001). Linear regression showed that ODT significantly depended on IRF (β = 0.61, P < 0.001), SRF (β = 0.34, P = 0.003), and scarring (β = 0.26, P = 0.050), while BCVA significantly depended only on scarring (β = −0.52, P < 0.001), and IRF (β = −0.36, P = 0.016).

**Conclusions:**

From dry AMD to active wet AMD, BCVA gradually worsened while ODT increased. The correlation between BCVA and ODT varied among these groups, indicating that AMD lesions affect them differently. ODT and BCVA should be used concurrently for better monitoring of the disease.

## Introduction

In age-related macular degeneration (AMD), the delicate structure of the choroidal vascular, retinal-pigmented epithelium (RPE), and neural retinal components are disrupted. Correspondingly, visual function deteriorates as the diseases progresses. It is generally thought to be the leading cause of blindness in people aged 50 and greater in developed countries. In America, over 6.5% of people aged 40 and over have been diagnosed with AMD [1], with 1.75 million of them in the advanced stage. This number is expected to reach 2.95 million by 2020 as the overall population ages [2].

Currently, intravitreal injection of ranibizumab or bevacizumab is accepted as the treatment of choice for wet AMD (also known as neovascularization AMD) [3–5]. To achieve the optimal treatment effect, it is essential to monitor retinal structural and functional changes in a timely manner during the initial treatment and follow-up visits [6]. For structural evaluation, non-invasive and high resolution optical coherence tomography (OCT) has been established as the method of choice. Central retinal thickness is the most straightforward summary indicator of retinal morphology. Best-corrected visual acuity (BCVA) is most often used to reflect the status of visual function.

However, these indices have certain limitations. Central retinal thickness only correlates reasonably well with BCVA shortly after the treatment begins in wet AMD. As treatment progresses, the correlation between BCVA and central retinal thickness becomes weak [7]. Recently, studies of AMD have shifted away from central retinal thickness to focus on more specific lesions, such as intra-retinal fluid (IRF), sub-retinal fluid (SRF), pigment epithelial detachment (PED), and scarring [8]. It was previously reported that BCVA correlates with IRF and scarring negatively and with SRF positively [8]. However, the reported correlation between BCVA and PED differs greatly among previous studies and the relationship between BCVA and other aspects of the retinal lesions remains unclear.

BCVA represents the resolution of the fovea region. It does not correlate well with the structural changes found using fundus fluorescein angiography and OCT [9–10]. Moreover, changes in BCVA often lag behind structural retinal changes [11]. Visual distortion is another important perception change that is reported by AMD patients. Many psychophysical tests have been developed to assess the perceived distortion in AMD [12]. Although the Amsler grid is simple to use and inexpensive, it lacks sensitivity and only provides a qualitative description of the perceived distortion [13]. Functional tests developed later, which include preferential hyperacuity perimetry (PHP), M-charts, and shape discrimination [14–16], can provide quantitative measurements of the distortion. However, none of those methods are firmly established or clinically routine. Recently, Bedell et al. developed a functional test based on the orientation discrimination threshold (ODT) that proved effective in identifying patients in the early stage of AMD [17].

In addition to measuring the changes of both BCVA and ODT, we also studied the retinal structural changes. The location and size of different types of lesions, such as drusen, PED, IRF, SRF, and scarring were quantitatively recorded with SD-OCT.

The aim of this study was to test whether specific tests of type of impairment of visual function, BCVA (resolution), or ODT (distortion), were more tightly associated with the location or the size of a specific lesion. Moreover, since these lesions reflect the status of inflammation [18–20], active or in resolution, the other aim of this study was to test the hypothesis if there is a hierarchy by which visual function is affected depending on the status of inflammation.

## Materials and methods

### Participants

From April 2015 through February 2016, 32 normal control subjects and 43 patients with AMD were recruited from the Eye Hospital of Wenzhou Medical University, Zhejiang, China. The study protocol was approved by the local ethics committee (Zhejiang Eye Hospital ethics committee) and adhered to the ethical tenets of the Declaration of Helsinki. All subjects signed the informed written consent before the test.

All of the AMD patients were diagnosed and carefully assessed by an experienced retina specialist (XL) through slit lamp microscope and a fundus imaging system. To account for the overlapped fundus features of ageing people and early AMD patients [21], a drusen larger than 63 microns in diameter was interpreted as a sign of dry AMD [22]. As such, ageing people whose drusen was smaller than 63 microns were excluded. Eyes with choroidal neovascularization (CNV) in the fovea were recruited as wet AMD [5]. In order to ensure the reliability of patient’s cooperation, patients with a BCVA less than 20/400 were excluded. Any patients with other retinal disease(s) that could affect BCVA were also not recruited into this study. Only one eye from each patient was enrolled. For patients with unilateral AMD, the affected eye was included. In cases of bilateral AMD and normal controls, the eye with better visual acuity was included. Detailed demographic and clinical information of the included patients are shown in the Results section.

The rationale behind our study is that visual function is highly correlated with retinal pathological status; for example, exudative alterations with underlying inflammatory processes involved in AMD [18–20]. Active inflammation can lead to severe interruption of structurally and functionally arranged neuronal networks, and resolution of inflammation could preserve some extent of function. Therefore, we hypothesized that there was a hierarchy by which visual function would be affected depending on the status of inflammation. As such, AMD patients were classified into three categories: 1) Active wet AMD (Wet-A). Patients with exudative macular alterations secondary to CNV lesion were classified as active wet AMD, which represents an exudative inflammatory status, with a rapid progression. 2) Non-active wet AMD (Wet-NA). Patients with CNV lesions but no evidence of exudative changes were classified as non-active wet AMD, indicating a state of resolution. 3) Dry AMD (Dry). Drusen is composed of not only extracellular wastes, but also the byproduct of local processes involving inflammation and complement system, which implies an inflammatory state. However, different from active CNV, this lesion develops rather slowly, and often remains non-exudative for a long time. [23]. Therefore, we assigned patients with drusen into the dry AMD group.

Eight AMD eyes (18.6%) were excluded because of accompanying severe cataract, leading to poor cooperation with the process of visual function tests. A total of 35 AMD patients (35 eyes) were recruited into the study. Among them, 21 subjects (51.2%) were men and fourteen (48.8%) were women. Their mean age was 65.9 ± 7.9 years. Five eyes (14.3%) were classified as Dry, 16 eyes (45.7%) were classified as Wet-NA, and 14 eyes (40.0%) were classified as Wet-A. A total of 32 persons (32 eyes) served as normal controls with 14 men (43.8%) and 18 women (52.6%). The average age of the normal subjects was 60.5 ± 5.2 years.

### Measurement of visual function

Early Treatment Diabetic Retinopathy Study (ETDRS) charts, (Precision Vision, La Salle, IL, USA), were used to measure each subject’s BCVA at 4 m. Metamorphopsia was first evaluated qualitatively with the Amsler grid that has black lines on a white background. The viewing distance was 30.5 to 35.6 cm (12–14 inches) and the subjects wore appropriate refractive correction for the near distance.

Metamorphopsia was further evaluated quantitatively with the orientation discrimination threshold (ODT) test. Specifically, stimuli were presented on a 14-inch (31.1 cm × 17.6 cm) laptop at a viewing distance of 1 m (Lenovo 430T, 1600 × 1200 pixels, 60 Hz) so that 1 pixel subtended 0.66 arcmin both horizontally and vertically. The program was written in MATLAB (MathWorks, Natick, MA) with the PTB3 version of the Psychophysics Toolbox [24–26]. The ODT was determined monocularly using an adaptive two-alternative forced-choice (2AFC) task, based on the ZEST algorithm [27–28]. The nontested eye was occluded with an opaque patch. At the beginning of each trial, the subject viewed the center of a 4°-diameter ring within which the stimuli were presented. After a button press, the ring disappeared and two stimulus patterns were presented sequentially with an interval of 1250 ms between them. The duration of each pattern was set at 50 ms to prevent possible saccadic intervention. The standard stimulus pattern consisted of four bright parallel lines, each of which was two pixels (1.3 minarc) wide, presented vertically on a dark background. Each line was randomly located within a central 4° annulus visual area, and the distance between two adjacent lines was at least 0.5° to assure no overlap. The line length was 0.4° if the visual acuity was 20/40 or better, and the line length was increased according to the following formula, if the visual acuity was worse than 20/40:

Line length = 0.3 + 0.05 * MAR (1)

**Fig 1:**
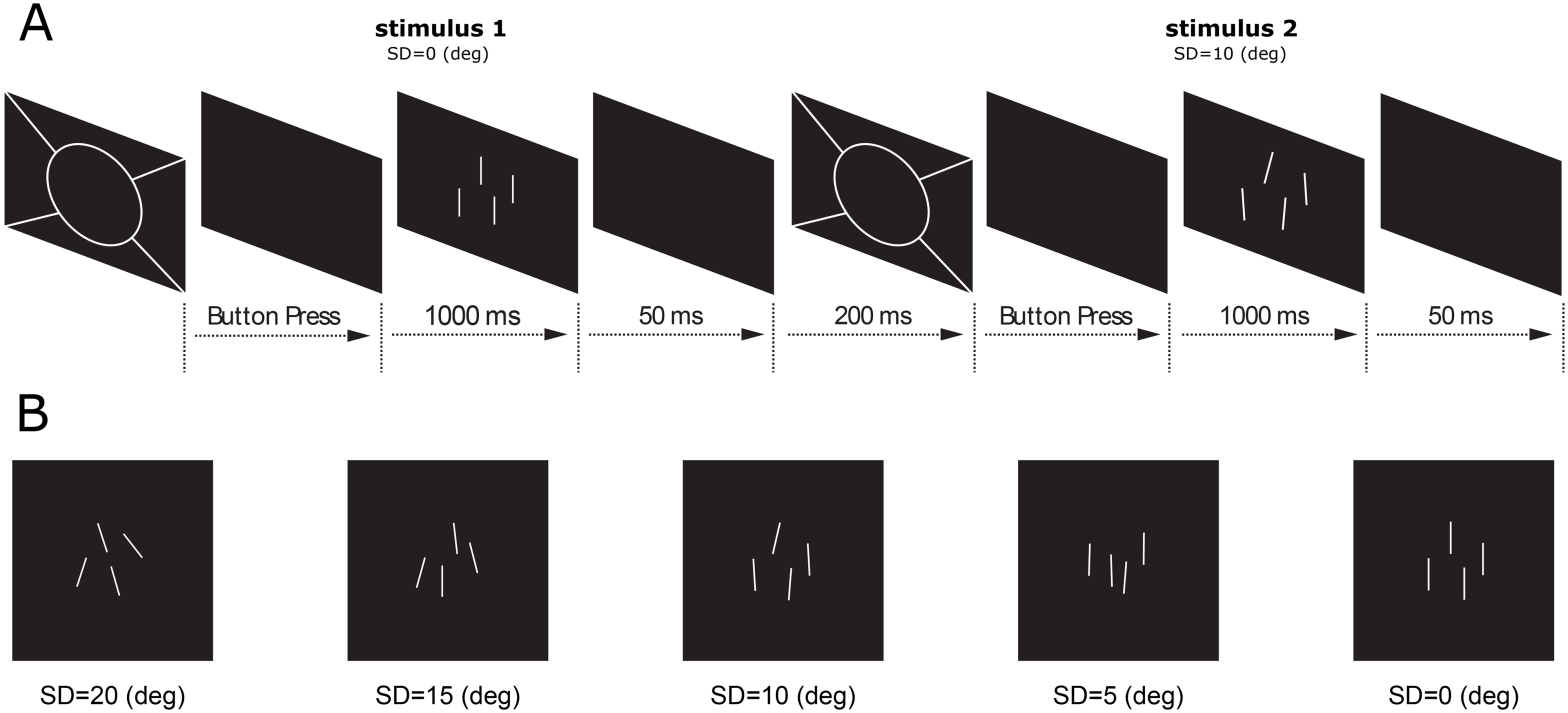
Stimulus-presentation of orientation discrimination threshold (ODT). **(A)** Stimulus-presentation sequence of ODT. **(B)** Demonstrations of stimuli with different standard deviations (SD, deg).

The line length was described as the visual angle (unit, degree). MAR was the minimum angle of resolution. The target stimulus had the same arrangement except that the orientations of the 4 lines were randomly deviated from vertical according to an angular standard deviation (SD). The order of the standard and target stimuli were randomized from trial to trial and after each trial, the subject had to decide which pattern contained the parallel vertical lines (Fig 1).

A Weibull function with slope at 3.5 was used as the likelihood function in the ZEST algorithm. The initial angular SD was set at 8° according to the average value from a previous study [17]. The probability density function (PDF) of the SD was automatically updated by multiplying the likelihood function with the prior PDF after each trial until the confidence interval of the PDF was less than 0.3 log units or a total of 30 trials were finished. The SD that was equal to the mean of the final PDF was defined as the orientation threshold. Testing of each eye required about 2 min to finish.

### Evaluation of structural changes from OCT images

**Fig 2:**
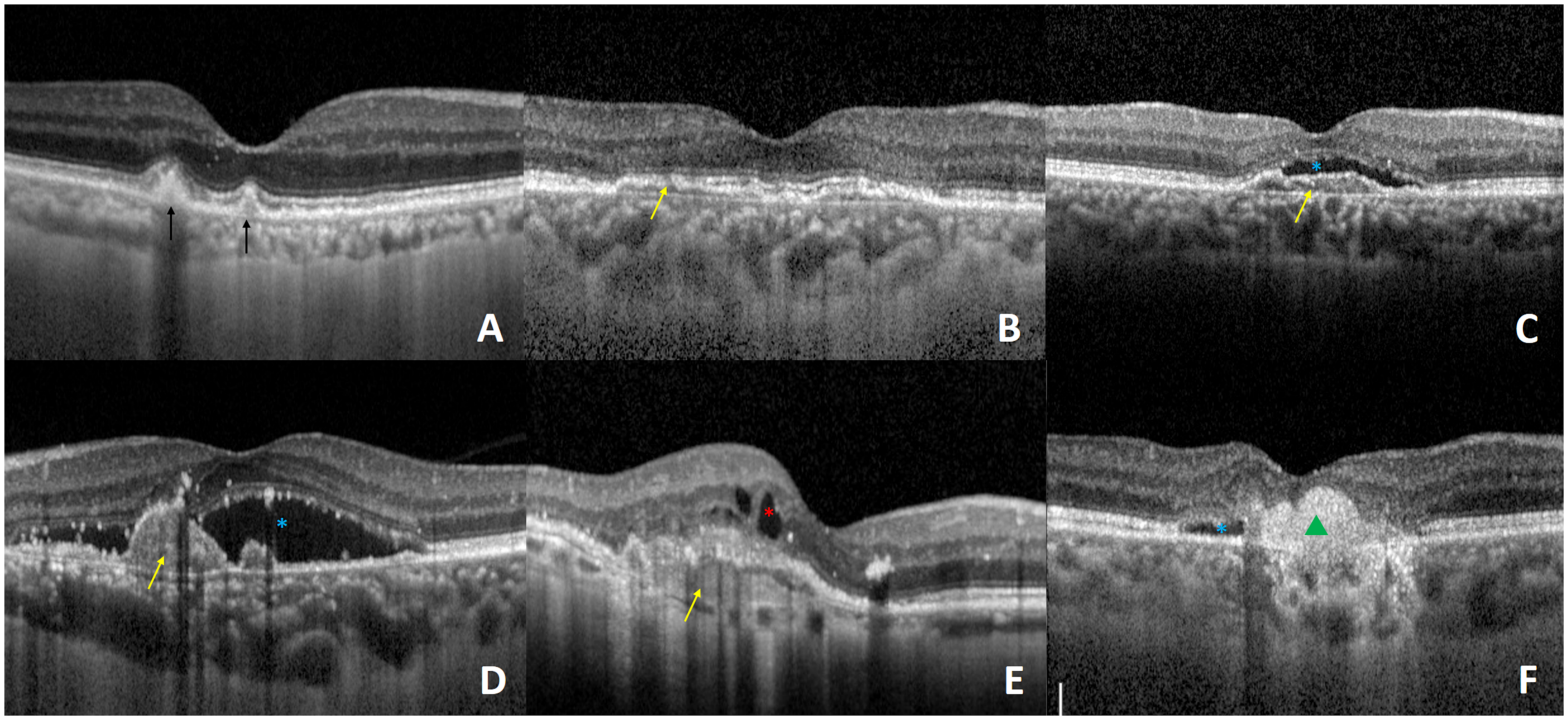
Optical coherence tomography (OCT) images show examples of lesions in different classifications of age-related macular degeneration (AMD). **(A)** Dry AMD. Dark arrows indicate drusens; **(B)** Non-active wet AMD, yellow arrow indicates a pigment epithelium detachment (PED); **(C-F)** Active wet AMD. Blue stars indicate sub-retinal fluid (SRF), the red star indicates intra-retinal fluid (IRF), and the green triangle indicates scarring.

Each eye with AMD was scanned using a Spectralis SD-OCT (Heidelberg Engineering, Heidelberg, Germany), and morphologic abnormalities in the macular region, including drusen, pigment epithelial detachment (PED), intra-retinal cystoid fluid (IRF), sub-retinal fluid (SRF), and scarring, were identified (Fig 2). Drusens were defined as discrete areas of RPE elevation over a hypo- or medium-reflective space. IRF appeared as round, minimally reflective spaces within the neurosensory retina, and SRF was defined as a nonreflective space between the neurosensory retina and the retinal pigment epithelium. PED was defined as a focal elevation of the reflective retinal pigment epithelium band over an optically clear or moderately reflective space that was either greater than 200 μm in height or more than 400 μm in width [29]. Scarring was characterized by well-demarcated, highly hyper-reflective material in the sub-retinal or sub-RPE space. An eye with AMD could have either one or multiple morphological abnormalities.

As mentioned before, the eyes with AMD were separated into three categories: dry AMD, non-active wet AMD, and active wet AMD. The classification was performed on the basis of OCT morphological characteristics. According to the diagnostic criteria of A-REDSR [22], an eye that had a drusen lesion larger than 63 microns was classified as dry AMD (Dry). If evidence of current or previous CNV lesions could be found in the macular region, the eye was classified as having wet AMD, which could be further classified as non-active AMD (Wet-NA) if only PED and/or scarring could be seen, or active AMD (Wet-A) if indicators of fluid leakage, such as SRF and/or IRF, were also present.

### Quantitative evaluation of morphological parameters

In the Wisconsin AMD grading system, the severity of AMD is assessed according to the location and size of the lesion (especially for drusens), presuming these two factors are vital to the patient’s outcome [30]. Considering this, we introduced three quantitative indexes in our study: size, location, and normalized effective area size (NEAS), which was derived from the integral calculation of both location and size.

**Fig 3:**
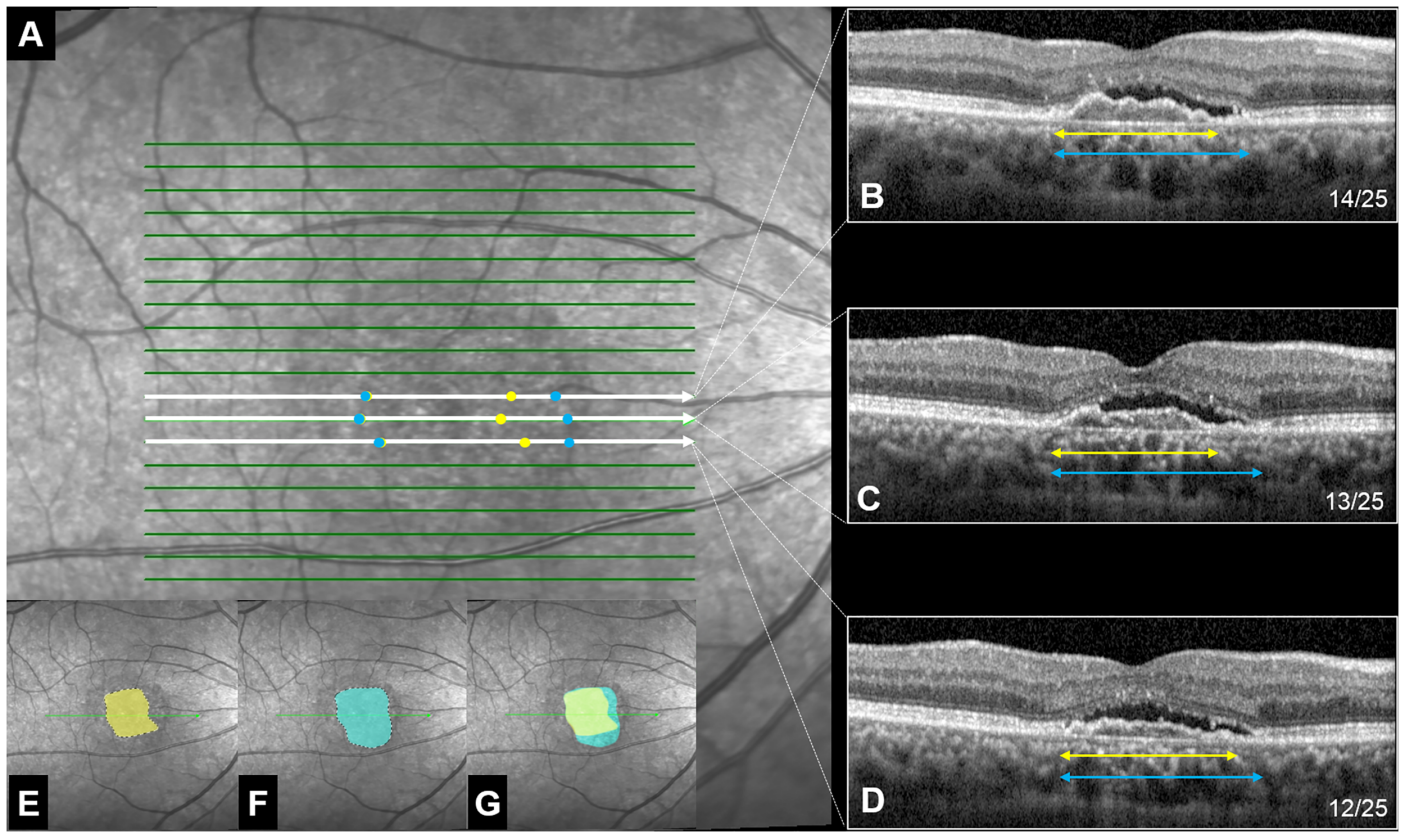
Optical coherence tomography (OCT) images illustrating the method to estimate the extent of abnormalities. Yellow = pigment epithelium detachment, PED; blue = sub-retinal fluid, SRF. **(A)** Surface view of the 25 scans (green lines). Three scans (12–14^th^, shown in white) are expanded in the 3 panels on the right side. **(B–D)** Cross sectional view of the 12–14^th^ scans in A. The yellow line indicates the range of PED and the blue line indicates the range of SRF. The arrow heads on the ends of the blue and yellow lines correspond to the blue and yellow dots in panel A. After this analysis was done for all 25 scan lines, all of the yellow dots were connected together to show the boundary of PED **(E)**, all of the blue dots to show the boundary of SRF and **(F)**, how the two types of abnormalities overlap on the retina **(G)**.

**Fig 4:**
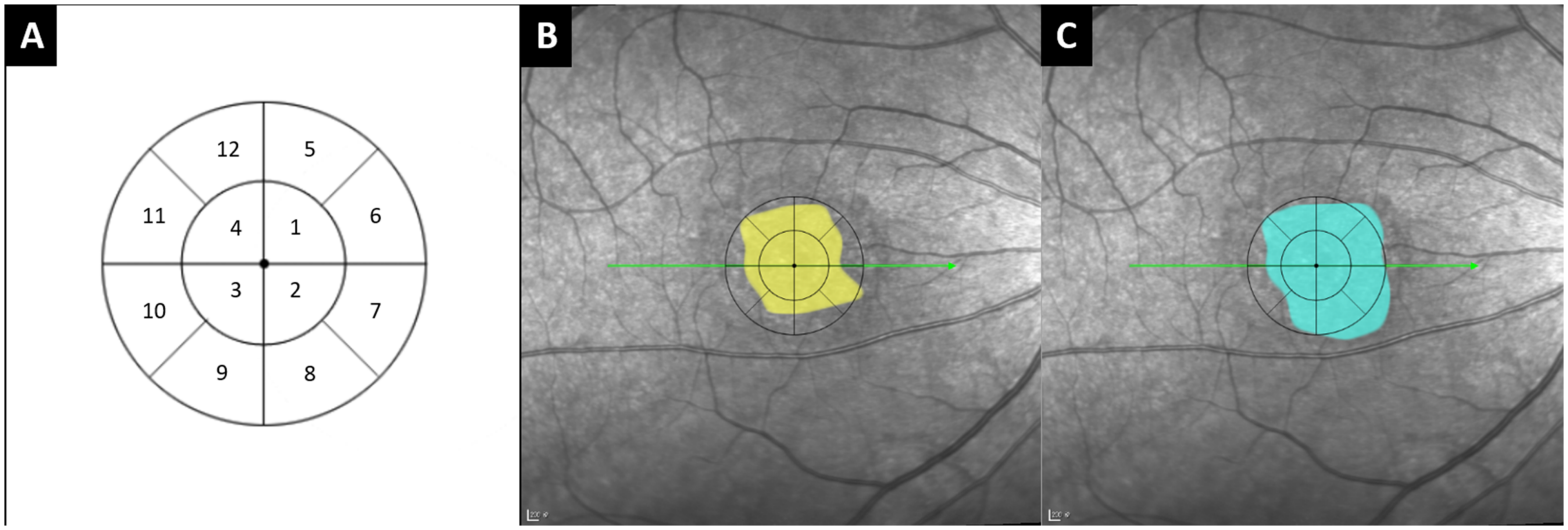
Method to calculate the normalized effective area size (NEAS) of each lesion. **(A)** A circular grid representing the macular region with the center at the foveola. The inner circle represents the central 4 deg and outer circle extends to the central 8 deg. Each sector of the inner circle (sectors 1–4) carries a weight of 1/6 and each sector of the outer circle (sectors 5–12) carries a weight of 1/24. As a whole, the central 8 deg had a weight of 1 (4 × 1/6 + 8 × 1/24 = 1). **(B)** Example of using the grid to estimate the NEAS of a pigment epithelium detachment lesion (PED, 0.833). **(C)** Example of using the grid to estimate the NEAS of a sub-retinal fluid lesion (SRF, 0.920). The images in panels B and C correspond to the lesions shown in Fig 3E and 3F.

**Size**: Commercial software (Adobe Photoshop, Version 12.0.4) was used to delineate the boundary of the lesion in every cross-sectional OCT image from the 25 consecutive scans in volume scan mode. The lesion area was determined by connecting together the extremities of the lesions in the single B scans and delineated manually using the Polygonal Lasso Tool (Fig 3). Once the area of the lesion was determined, a circular grid was placed with its center at the fovea (Fig 4). The inner circle represented the central 4 degree visual angle and outer circle extended to the central 8 degree. The size of the lesion was calculated based on the sum of pixels selected regardless of location, and then scaled to the relative size of the outer circle. **Location**: Because the inner subfields nearest the fovea are more important for visual function compared to the outer ones, the lesions in the inner subfields were quantified as four for the facility of analysis; whereas, those in the outer ring were quantified as one. **Normalized effective area size (NEAS)**: For parallel assessment of location (weight ratio of inner to outer area is 1:4), we assigned each inner subfield a weight of 1/6 (0.167) and each outer subfield a weight of 1/24 (0.042) (Fig 4). The normalized effective area size (NEAS) of the lesion was calculated as follows:

NEAS = Σ size*weight (2)

To exclude individual biases, readings were performed by two different retinal physicians independently. The intra-class correlation coefficient (ICC) revealed high agreement between the measurements by the two experts.

### Statistical analysis

Analysis of variance (ANOVA) was used to compare the differences in BCVA and ODT among normal eyes and eyes with different classifications of AMD. Pearson correlation and partial correlation analysis was applied to examine the relationship between quantitative parameters. Multiple linear regression was used to evaluate the relationships between morphological parameters and visual functions. All of these analyses were performed using the R programming package (version 3.3.1). P ≤ 0.05 was defined as statistically significant.

## Results

### Structural changes

#### Lesions identified in the OCT scan

Lesions identified in the OCT scans were classified into the following categories: drusen, PED, SRF, IRF, and scarring. For each lesion, the location, size, and normalized effective area size (NEAS) were calculated from OCT data obtained with the volume scan mode. The information about the specific types of lesion and their location, size, and NEAS in each eye can be found in Table 1.

**Table 1:** Demographic data, macular lesions, and their locations, size, and associated normalized effective area size (NEAS) of age-related macular degeneration (AMD) patients with different classifications.

Classification	ID-Sex-Age	Lesion [Location, Size, NEAS]				
		Drusen	PED	IRF	SRF	Scar
**Dry**	1-M-82	[4, 0.386, 0.150]	-	-	-	-
	2-M-75	[4, 0.230, 0.441]	-	-	-	-
	3-F-67	[4, 0.022, 0.057]	-	-	-	-
	4-M-69	[4, 0.039, 0.059]	-	-	-	-
	5-M-70	[4, 0.016, 0.042]	-	-	-	-
**Wet-NA**	6-M-61	-	[4, 0.100, 0.259]	-	-	-
	7-F-61	-	[4, 0.042, 0.110]	-	-	-
	8-M-64	-	[4, 1.382, 1.000]	-	-	-
	9-M-53	-	[4, 0.169, 0.263]	-	-	-
	10-F-59	-	[4, 0.442, 0.482]	-	-	-
	11-M-55	-	[4, 1.115, 0.622]	-	-	-
	12-M-56	-	[4, 0.122, 0.275]	-	-	-
	13-F-55	-	[1, 0.051, 0.044]	-	-	-
	14-M-67	-	[4, 0.704, 0.454]	-	-	-
	15-F-78	-	[4, 1.354, 0.834]	-	-	-
	16-M-68	-	[4, 0.465, 0.684]	-	-	-
	17-F-62	-	[4, 0.362, 0.492]	-	-	-
	18-M-57	-	[1, 0.144, 0.029]	-	-	-
	19-F-55	-	[1, 0.095, 0.028]	-	-	-
	20-F-56	-	[4, 0.029, 0.074]	-	-	-
	21-F-67	-	[4, 1.989, 0.943]	-	-	-
**Wet-A**	22-M-67	-	[4, 0.726, 0.359]	-	[4, 2.164, 1.000]	-
	23-F-65	-	[4, 0.346, 0.400]	-	[4, 0.800, 0.323]	[4, 0.191, 0.313]
	24-M-59	-	[1, 0.055, 0.047]	[1, 0.010, 0.004]	-	-
	25-F-76	-	[4, 1.698, 1.000]	[1, 0.092, 0.021]	-	-
	26-M-65	-	[4, 0.156, 0.327]	-	[4, 0.014, 0.036]	-
	27-M-70	-	[4, 0.055, 0.077]	[4, 0.540, 0.395]	[4, 0.180, 0.401]	-
	28-M-78	-	[4, 1.369, 0.447]	[4, 0.146, 0.185]	-	-
	29-M-68	-	[4, 0.638, 0.833]	-	[4, 0.939, 0.920]	-
	30-M-82	-	[4, 0.665, 0.378]	-	[4, 1.343, 0.867]	-
	31-M-64	-	-	[4, 0.399, 0.406]	-	[4, 0.699, 0.447]
	32-F-72	-	[4, 0.119, 0.190]	-	[4, 0.964, 0.944]	-
	33-M-72	-	[4, 4.228, 1.000]	[4, 0.098, 0.198]	[1, 1.982, 0.035]	-
	34-F-66	-	[4, 0.188, 0.124]	[4, 0.307, 0.225]	-	-
	35-F-65	-	-	[4, 0.112, 0.240]	-	[4, 0.547, 0.646]

Dry: dry AMD; Wet-NA: non-active AMD; Wet-A: active AMD; NEAS: normalized effective area size; PED: retinal pigment epithelium detachment; SRF: sub-retinal fluid; IRF: intra-retinal fluid; -: not available.

### Functional changes

Compared with normal controls, visual acuity and metamorphopsia (AMSLER grid, and orientation discrimination) in AMD patients had clearly deteriorated. Among these patients, as expected, these two visual functions were strongly associated with their inflammatory status. Their acuity decreased progressively from Dry and Wet-NA to Wet-A AMD.

#### Results of the Amsler grid test

Perceived distortion on the Amsler grid was reported in 42.9% (15/35) of the eyes with AMD. The proportion of eyes with reported distortions was 20.0% (1/5) in dry AMD, 56.3% (9/16) in Wet-NA AMD, and 35.7% (5/14) in Wet-A AMD. No subjects in the normal group reported abnormalities on the Amsler grid test. There were no significant differences among the proportions from different groups (P = 0.300, Fisher exact test).

#### ODT and BCVA

**Fig 5:**
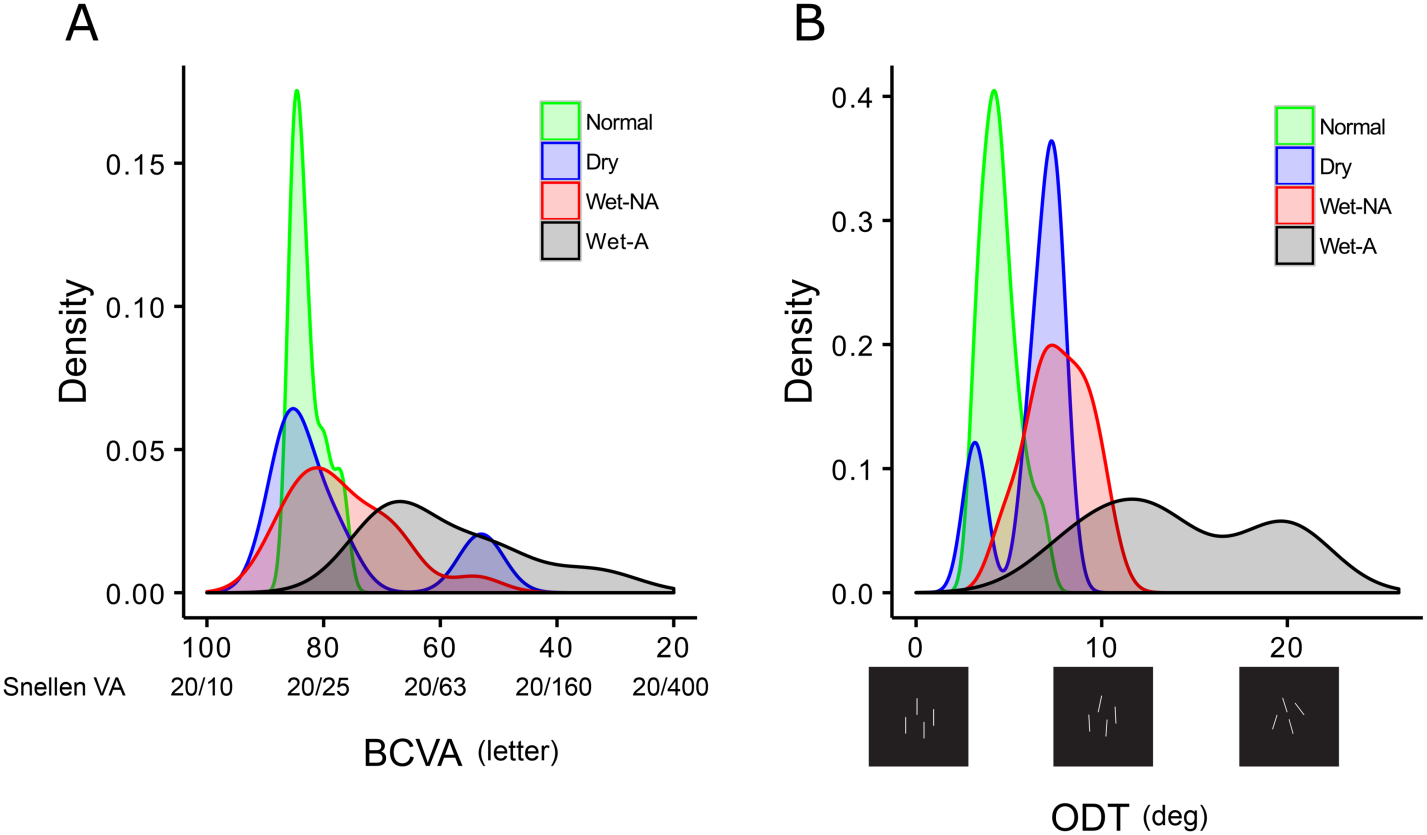
Smoothed probability density plots showing the distribution of best-corrected visual acuity (BCVA) (A) and orientation discrimination threshold (ODT) (B).

BCVA decreased across the groups with AMD, with mean BCVA values 82.7 ± 2.8, 77.6 ± 14.2, 76.7 ± 9.1, and 58.4 ± 13.2 ETDRS letters for the normal, Dry, Wet-NA, and Wet-A groups, respectively (Fig 5-A). Statistically, the values in the Wet-NA group were significantly lower than in the normal group (Tukey test, P < 0.001). The values in the Wet-A group were also significantly lower than those in the Dry and Wet-NA groups (Tukey test, P < 0.001, P < 0.001 respectively). One patient in the Dry group (1/5 = 20.0%), 7 patients in the Wet-NA group (7/16 = 43.8%), and 14 patients in the Wet-A group (14/14 = 100%) had a BCVA that was lower than the lower limit of the 95% confidence interval (mean ± 2 SD) for the normal subjects.

Orientation discrimination thresholds also became more impaired across the AMD groups, with mean ODT values of 4.5 ± 1.0, 6.3 ± 1.8, 7.7 ± 1.7, and 14.3 ± 4.7 deg for the normal, Dry, Wet-NA, and Wet-A groups, respectively (Fig 5-B). Statistically, the thresholds in both the Wet-NA and Wet-A groups were significantly higher than in the normal group (Tukey test, P < 0.001 and P < 0.001 respectively). The values in the Wet-A group were also significantly higher than in the Dry and Wet-NA AMD groups (Tukey test, P < 0.001 and P < 0.001 respectively). Three patients in the Dry group (3/5 = 60.0%), 13 patients in the Wet-NA group (13/16 = 81.3%), and 14 patients in the Wet-A group (14/14 = 100%) had an ODT that fell outside of the 95% confidence interval (mean ± 2 SD) for the normal subjects.

#### The correlation between ODT and BCVA

**Fig 6:**
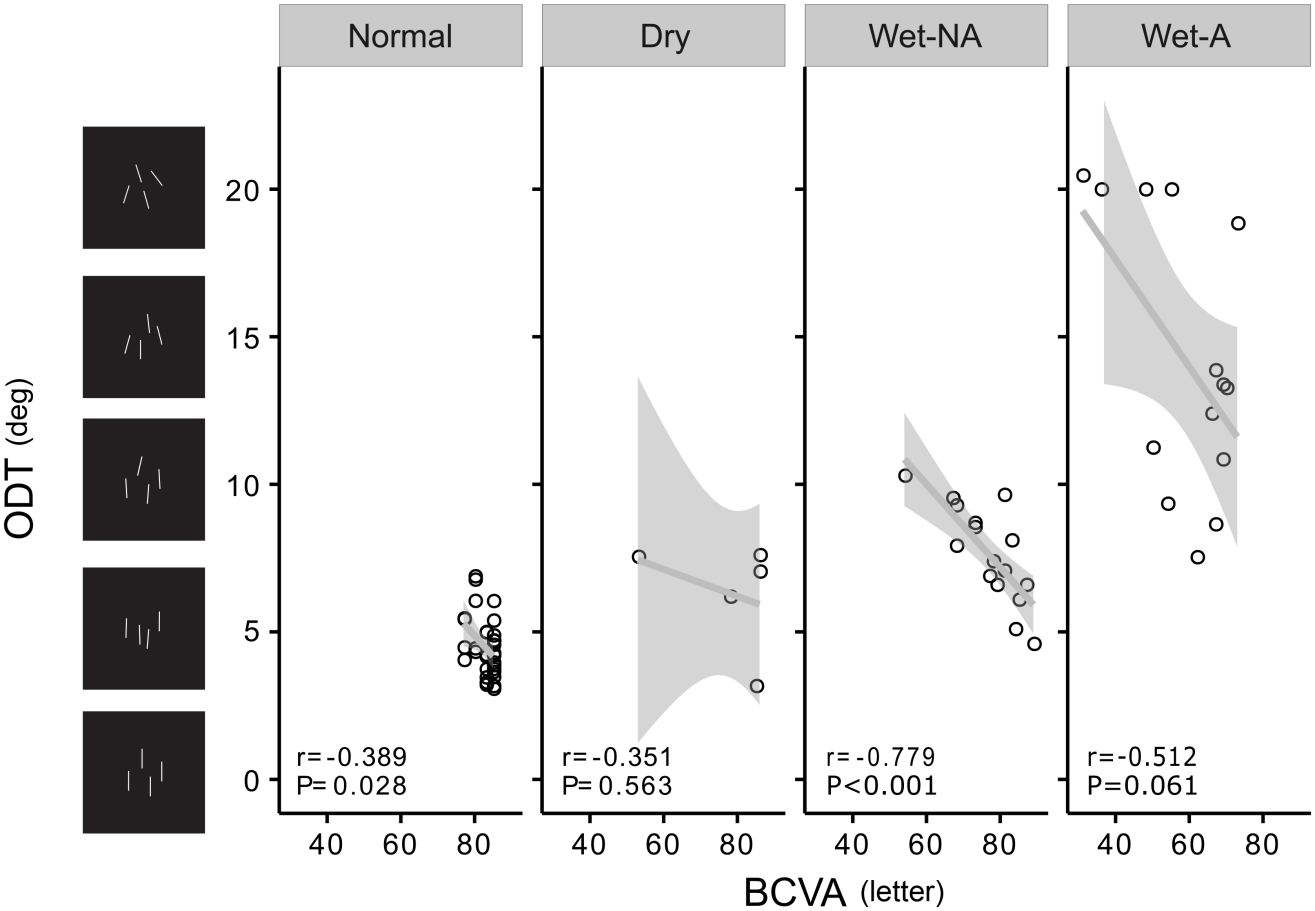
Relationships between best-corrected visual acuity (BCVA) and orientation discrimination threshold (ODT) in patients with different classifications of age-related macular degeneration (AMD).

The correlation coefficients between ODT and BCVA were −0.39, −0.35, −0.78, and −0.51 in the normal, Dry, Wet-NA, and Wet-A AMD groups, respectively (Fig 6). ODT and BCVA showed a significant correlation in the normal and Wet-NA AMD groups (P = 0.028, < 0.001 respectively). Considering that both ODT and BCVA are functional measurements that depend on structural changes in the retina, we further performed a partial-correlation analysis between OCT and BCVA using the groups of AMD as the mediator (normal = 0, Dry = 1, Wet-NA = 2, Wet-A = 3). The partial correlation between ODT and BCVA was moderate at most (r = −0.61, P < 0.001), suggesting that BCVA and ODT are measuring different functional aspects of the visual system.

### The association between functional and structural changes

**Fig 7:**
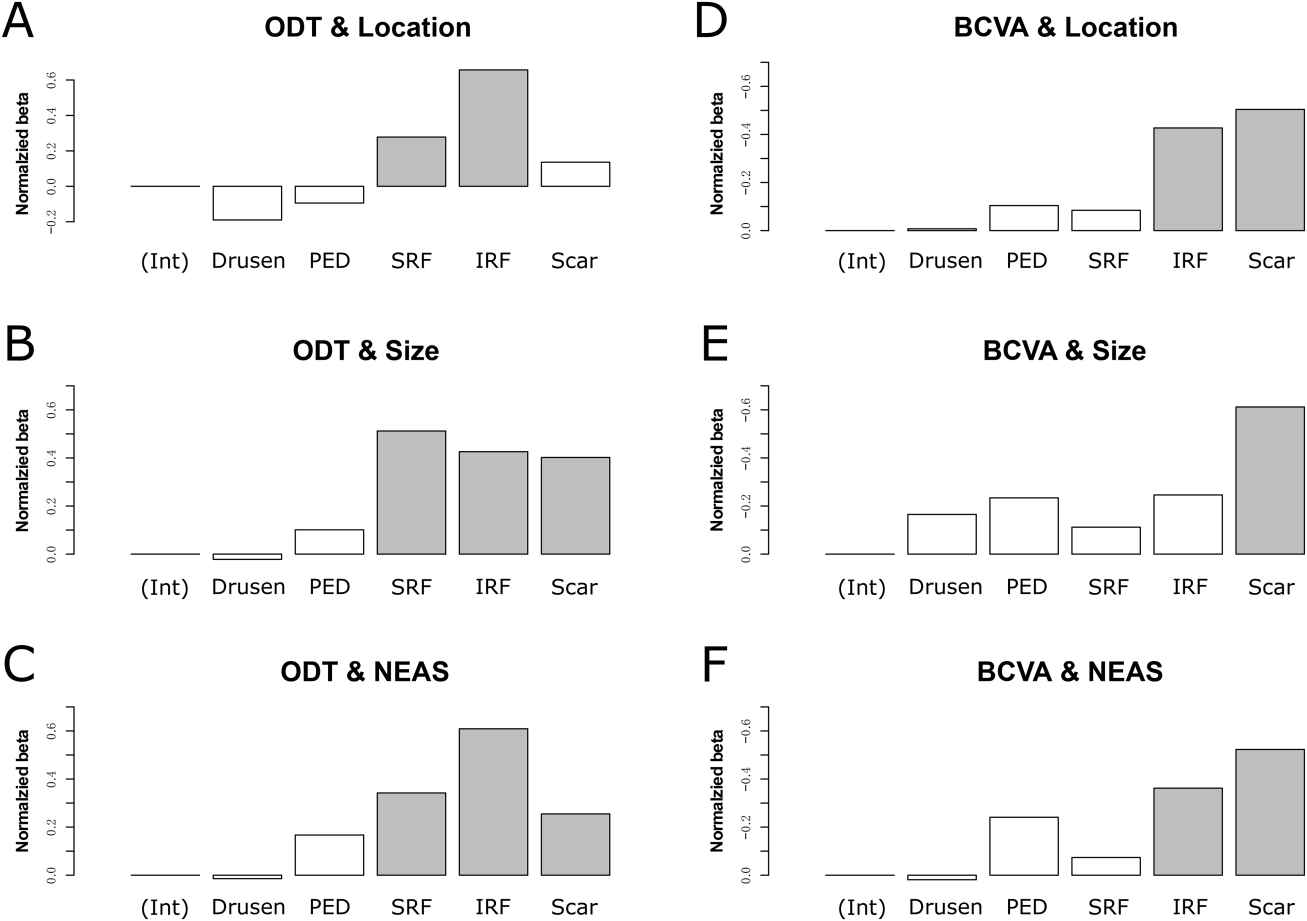
Normalized multivariate-regression weights of different types of lesions for the variation of orientation discrimination threshold (ODT) and best-corrected visual acuity (BCVA). Normalized weights of lesions’ locations **(A)**, sizes **(B)**, and NEAS **(C)** for ODT; normalized weights of lesions’ locations **(D)**, sizes **(E)**, and NEAS **(F)** for BCVA. A filled bar indicates a significant contribution to the regression model.

We performed multivariate regression of the two functional measurements on the location, size, and NEAS of macular lesions, which included drusen, PED, SRF, IRF, and scarring. For ODT, the analysis of the location identified IRF and SRF as significant factors with IRF as the dominant one. However, the analysis of size also identified scarring as a significant factor but SRF was the most important factor (Fig 7A and 7B). A similar discrepancy existed for BCVA. Location analysis revealed both scar and IRF as significant factors. However, for the size analysis, only scarring was significant (Fig 7D and 7E). Therefore, it is essential to perform a comprehensive assessment (NEAS) combining the contributions from both location and size. The analysis of NEAS revealed that BCVA and ODT depend on lesion types differently (Fig 7C and 7F). Scarring had the strongest influence on BCVA (β = −0.52, P < 0.001), followed by IRF (β = −0.36, P = 0.016). For ODT, IRF had the strongest influence (β = 0.61, P < 0.001), followed by SRF (β = 0.34, P = 0.003) and scarring (β = 0.26, P = 0.050).

## Discussion

Our study has produced important findings on three aspects. First, for both BCVA and ODT, the impairment of function followed the hierarchy of inflammation status. The function loss was least in the cases with dry AMD. The functional loss in cases with active inflammation, namely Wet-A AMD, was much greater than that in cases having inflammation with resolution status. Second, between BCVA and ODT, the correlation between BCVA and ODT was significant in the Wet-NA group but was non-significant in other categories. With the classification of AMD as a mediator, a weak, but significant, partial correlation between BCVA and ODT was identified. Third, it is important to take both the location and size of the lesions into consideration when assessing the link between functional impairment and lesions. Regression analysis showed that scarring exerts the largest effect on BCVA, whereas ODT is associated most strongly with accumulated fluid in the retina, such as IRF and SRF.

### Association between visual function and different types of lesions

Our results showed that IRF impairs both BCVA and ODT significantly. This agrees with previous study findings [7, 31]. Several factors may contribute to this long-lasting disrupting effect. First, in IRF, fluid often accumulates between Henle’s fibers or spreads into the inner nuclear layer [32]. The existence of a fluid cyst also can squeeze the Henle’s fibers, thereby distorting the normally orderly mosaic of photoreceptors [33]. Second, IRF may be caused by an abnormal water metabolic process inside of Müller cells [32]. Because retinal neurons work closely with Müller cells as a unit for metabolism and signal transduction and transmission, edema in the Müller cells may cause disruption of signaling by retinal neurons. Third, light that traverses the optical pathway within the eye encounters the cyst before it reaches the photoreceptors. The refractive index of the fluid in the cyst is different from the surrounding retinal tissues and this inevitably increases aberrations within the image formed at the retinal photoreceptor layer [34]. In contrast, other lesions, such as SRF and PED, are located posterior to the photoreceptors along the optical pathway.

SRF has little effect on visual acuity, but has a large effect on perceptual distortion. This outcome is consistent with previous studies. Sharma reported a positive association in patients with AMD between SRF and visual acuity over 2 years of follow-up [31]. A similar finding was found in another double-masked clinical trial that investigated the efficacy and safety of VEGF treatment for AMD [8]. In studies of central serous chorioretinopathy and branch retinal vein occlusion, SRF was found to be correlated with visual distortion [35–36].

The exact mechanism by which SRF exerts different effects on BCVA and ODT is still not clear. Because SRF causes a separation between the neural retina and RPE, the photoreceptors in the affected region will be distributed along the sloping sub-retinal space instead of on a flat surface [34]. In addition, SRF can cause the inter-photoreceptor spacing to increase, such that an image in an area with SRF will be registered on fewer photoreceptors than in the surrounding retina. These local variations in image registration might cause an uneven cortical representation for different parts of the retinal image and lead to visual distortion [37].

Retinal scarring was found to affect both visual acuity and visual distortion in our study. Its severe effect on these visual functions could come from the following several aspects. First, scarring severely damages the normal retina structure. Photoreceptors are greatly reduced in number and the outer nuclear layer is often disrupted in the region of a scar [38]. Second, sub-retinal hyper-reflective material (SRHM) and fibrosis, which precede scarring, can disturb the normal retinal structure and lead to visual impairment [39–40]. Third, since scarring often appears in patients who have received multiple anti-VEGF injections, one cannot rule the possibility of potential damage directly caused by anti-VEGF administration. Suzuki et al. reported that VEGF has a protective effect on photoreceptors, and anti-VEGF often works against this protective effect [41]. In addition, Brar et al. reported that anti-VEGF can neutralize the protective effect of VEGF on ganglion cells [42].

### ODT vs. visual acuity

In the patients with Wet-NA AMD, the most common types of lesions were PED, which showed similar associations with BCVA and ODT. This might explain why BCVA and ODT have a relatively high correlation in the Wet-NA group. In the Wet-A group, the relationships between SRF, IRF, and scarring on ODT and BCVA are substantially different; therefore, a low correlation between them is understandable. The varying correlations between BCVA and ODT in different AMD categories indicate that they represent two different aspects of visual function and they should be used simultaneously in the care of AMD. Paying attention only to visual acuity may lead to missing important functional changes in these patients. For example, in our study, we found that SRF does not affect visual acuity as much as it affects ODT. This provides new insights into the strategy used for AMD management. One often-debated issue is whether treatment should be continued or stopped when a small amount of SRF persists [31]. The authors of the CATT study reported that the presence of SRF was surprisingly associated with a better VA at year 2, while other studies reported a contrary result [43]. However, because there is a strong association between SRF and visual distortion, stopping treatment of SRF does not seem wise.

### ODT vs. Amsler grid test

The Amsler grid is the most commonly used method to detect visual distortion. However, it has been shown to be insensitive in our research and in other studies [44]. This might be due to the “filling-in” phenomenon that occurs in the visual cortex [45]. When the signal from certain regions of the retina are missing, the visual cortex utilizes the signals from neighboring areas to construct a cortical representation of the silent area of the retina [46]. The repetitive and continuous horizontal and vertical lines of the Amsler grid make the “filling-in” process much easier. Because of the scanning eye movements over a static Amsler grid, the distorted signals from an affected retinal region can be averaged with the normal signals arising from adjacent healthy retina, presumably resulting in a reduction of the perceived distortion. In the ODT test, however, each visual stimulus is only present for 50 ms, which minimizes the “average effect.”

The present study has some limitations. Although this design gave us the opportunity to study the effects of many different types of retinal lesions, it lacks a comparison between retinal structure and function before and after the anti-VEGF treatment. Some of the patients in the study had already received medical treatment for AMD. By the time they were included in the study, some of their lesions, e.g. SRF or IRF, had already receded or disappeared. Whether these previous lesions exert any long-lasting residual effects on visual function remains unknown. In the future, a longitudinal study will be designed to address these issues.

In conclusion, BCVA and ODT represent two different aspects of visual function. In patients with AMD, BCVA is mainly affected by scarring and IRF, and ODT is mainly affected by IRF, SRF, and scarring. Applying the two functional indices of BCVA and ODT concurrently should improve the efficacy of monitoring the progress of patients with AMD.
